# Changes in the levels of inflammatory markers after transthoracic device closure of ventricular septal defects in pediatric patients

**DOI:** 10.1186/s13019-019-0900-4

**Published:** 2019-04-08

**Authors:** Jiang-Shan Huang, Qiang Chen, Liang-Wan Chen, Yur-Ren Kuo, Zhi-Nuan Hong, Hua Cao

**Affiliations:** 1 Department of Cardiac Surgery, Fujian Provincial Maternity and Children’s Hospital, Affiliated Hospital of Fujian Medical University, the Daoshan road 18, Gulou District, Fuzhou, 350001 People’s Republic of China; 2 Department of Cardiovascular Surgery, Union Hospital, Fujian Medical University, Fuzhou, 350001 People’s Republic of China; 3 Division of Plastic Surgery, Department of Surgery, Kaohsiung Medical University Hospital, 100 TzYou 1st Rd, Kaohsiung City, 80756 Taiwan

**Keywords:** Systemic inflammatory response syndrome, Ventricular septal defect, Surgery, Hybrid procedure

## Abstract

**Background:**

Transthoracic device closure of ventricular septal defect (VSD) is widely used in the clinic, especially in China. Changes in inflammatory marker levels after transthoracic device closure of VSD in pediatric patients have not been reported.

**Methods:**

We retrospectively collected clinical data for 85 pediatric patients in our hospital from September 2017 to January 2018. The patients were divided into two groups according to treatment (device group vs. surgical group). The clinical and experimental data from the two groups were statistically analyzed.

**Results:**

Clinical outcomes were good in all patients without any fatal complications. Similar increasing trends in inflammatory markers (white blood cell (WBC) count, procalcitonin (PCT), C-reactive protein (CRP), and interleukin-6 (IL-6)) were found in the two groups, both of which showed noticeable systemic inflammatory responses. In addition, no significant difference in the postoperative levels of inflammatory markers was observed between these two groups.

**Conclusions:**

Although transthoracic device closure of VSD seems to be less traumatic and involves a quicker recovery, it also induces a systemic inflammatory response as measured by WBC count and PCT, CRP and IL-6 levels, and the altered trends in inflammatory markers were similar to those of conventional surgery under CPB.

## Background

Ventricular septal defect (VSD) is the most common type of congenital heart disease (CHD) [1, 2]. The gold standard of treatment is surgical repair under cardiopulmonary bypass (CPB) [3], but this procedure is associated with a visible scar, long postoperative hospital stay and probable thoracic deformation [4]. In recent years, transthoracic device closure of VSD has been widely used, especially in China. Previous reports have described its minimal invasiveness, rapid recovery, low cost and wide indication [5–7]. Studies have explored the elevation in inflammatory marker levels after cardiac surgery under CPB [8–10], but little attention has been given to whether postoperative levels of inflammatory markers are altered by transthoracic device closure of VSD. Currently used clinical inflammatory markers include white blood cell (WBC) count, C-reactive protein (CRP), procalcitonin (PCT), and interleukin-6 (IL-6). In addition to clinical manifestations, these levels of inflammatory markers also represent important criteria for evaluating the severity of the systemic inflammatory response [8–10]. Therefore, changes in the levels of these inflammatory markers during the perioperative period were examined to determine whether transthoracic device closure of VSD results in a systemic inflammatory response and to assess the severity of this response.

## Materials and methods

In this retrospective study, we collected the medical records of 88 consecutive pediatric patients with VSD in our department from September 2017 to January 2018. Furthermore, all patients received treatment from the same experienced surgical team. All patients were diagnosed with isolated perimembranous VSD and were sufficiently assessed by transthoracic echocardiography (TTE). Routine preoperative examinations, including chest radiography, electrocardiogram, TTE, complete blood count, biochemistry and measurement of inflammatory markers (which included CRP, PCT and IL-6), were performed for all patients. No clinical or laboratory signs of infection were noted before the procedure.

The VSD closure guidelines were consistent with those of previous reports [5–7]. The inclusion criteria were as follows: no other intracardiac malformation and/or organ disease; a significant hemodynamic left-to-right shunt and/or chamber enlargement and capacity overload and/or mild to moderate pulmonary hypertension; and no aortic regurgitation. The exclusion criteria were age under 9 months, weight below 6 kg, pulmonary or other organ infection, severe pulmonary hypertension or Eisenmenger syndrome. Patients with significantly increased preoperative inflammatory markers (WBC count, PCT, CRP, and IL-6) and with preoperative or postoperative infection were also excluded from this study. We defined the criteria for infection as follows: 1, body temperature higher than 38.5 °C; 2, chest radiography indicated pulmonary infection; 3, pulmonary auscultation can detect obvious moist rales; 4, the expectorated sputum was a clearly purulent sputum; and 5, bacteria appeared in sputum and blood cultures [11]. All of the above data were recorded in detail in the medical records. The final diagnosis of infection was made independently by two experts, and a third researcher evaluated whether specific cases were included or excluded.

When we retrospectively analyzed the data, we excluded 3 patients from the study because they had postoperative pulmonary infections. The remaining 85 patients we selected did not show any noticeable clinical or laboratory signs of infection after their procedures. The patients were divided into two groups according to the different VSD closure procedures. Thirty-eight patients with isolated and restrictive VSD (VSD size ranging from 4 to 6 mm) in group A underwent transthoracic device closure. The 47 patients in group B underwent surgical repair under CPB. This group included patients with nonrestrictive or misaligned VSD, failure to complete or refusal of device closure, and ineligibility for device closure. In this group, 21 patients were selected to receive a right infra-axillary incision, and 26 patients were selected to receive a median incision based on their individual situations. A successful VSD closure was defined as no large residual shunt (< 2 mm). The clinical features of the two groups are shown in Table 1. No statistically significant differences in gender or age distribution were found between the two groups.

**Table 1:** Preoperative data comparison between two groups of patients

Item	Group A	Group B	*p* value
N	38	47	
Gender(M/F)	21/17	24/23	
Age(years)	1.7 ± 1.2	1.4 ± 1.4	0.24
Weight(kg)	9.1 ± 2.3	9.3 ± 2.3	0.72
Pulmonary hypertension (mm Hg)	36.3 ± 8.2	37.2 ± 5.2	0.55
Size of VSD(mm)	4.8 ± 1.2	5.3 ± 1.3	0.17
Cardiothoracic ratio	0.53 ± 0.1	0.56 ± 0.1	0.41

The transthoracic device closure of VSD without CPB in group A was performed under general anesthesia. In our cardiac center, a lower partial median sternotomy was performed, and the sternum was partially spread to expose the operative fields. Then, the pericardium was opened and suspended to expose the free wall of the right ventricle. With the guidance of transesophageal echocardiography (TEE), the transport track was set up and traversed the right ventricle to the VSD to the left ventricle. The occluder was inserted into the sheath and advanced into the left ventricle by the push rod, and then the two dishes of the occluder were released in turn into the two sides of the ventricular septum to close the VSD guided by TEE. The occluder was released from the push rod once its correct location was confirmed. Subsequent treatment included three months of anticoagulant therapy. We have described many of these procedures in our previous reports [12].

In group B, the surgical procedure was performed under general anesthesia. Two kinds of surgical incisions were used in this study: the traditional median incision and the right infra-axillary incision. Regardless of the incision type, the surgical field (which at minimum included the ascending aorta root, right atrium, partial right ventricle, and upper and inferior vena cava) was fully exposed. After systemic heparinization, a routine CPB was established. Then, the ascending aorta was clamped, and cardioplegia was injected at the root of the aorta. After cardiac arrest, the right atrium was opened, and the ventricular septal defect was repaired with a patch through the tricuspid valve.

We retrospectively analyzed the levels of the selected inflammatory markers, which were collected on the first preoperative day (preOP) and the first, second, third, fifth and seventh postoperative days (POD1, POD2, POD3, POD5 and POD7, respectively), if necessary. In addition, we also collected and analyzed data on the postoperative mechanical ventilation time, duration of time in the intensive care unit (ICU), and length of hospital stay.

Continuous data are presented as the means±SD. We used Tukey’s HSD (honest significant difference) test to compare the data collected at different time periods using analyses of variance and repeated measures data. A statistically significant difference was defined as a *p* value less than 0.05.

## Result

On the basis of the inclusion and exclusion criteria, 85 pediatric patients were included in this study. All patients received a successful closure without reoperation for VSD, cerebrovascular accident, multiple organ dysfunction, or any serious postoperative complications, including severe low cardiac output syndrome, death, malignant arrhythmia or complete atrioventricular block.

Group A had a shorter operative time, postoperative mechanical ventilation time, duration of intensive care and length of incision than group B. The corresponding data are shown in Table 2.

**Table 2:** Perioperative and postoperative data comparison between two groups of patients

Item	Group A	Group B	*p* value
Operation time(min)	24.6 ± 7.6	108.5 ± 23.0	*p* < 0.01
Mechanical ventilation time(h)	2.9 ± 2.1	10.3 ± 4.7	*p* < 0.01
Intensive care unit time(h)	5.7 ± 3.8	16.7 ± 3.9	*p* < 0.01
Drainage(ml)	12.5 ± 10.3	24.2 ± 15.7	*p* < 0.01
The incision length(cm)	2.8 ± 0.7	7.5 ± 1.2	*p* < 0.01

The values of the inflammatory markers that were selected for group A and group B on the first preoperative day and the first, second, third, fifth and seventh postoperative days (if necessary) are shown in Table 3. The usual length of hospitalization for these groups was 3–6 days, and subsequent examinations were completed in the outpatient department. As shown in this table, we found that WBC count and levels of CRP, PCT and IL-6 were significantly increased after both treatments. The changes in CRP and IL-6 were similar in the two groups, as CRP levels peaked on the second postoperative day (POD2) in both groups, while IL-6 levels peaked on the first postoperative day and then began to decrease. WBC count and PCT levels peaked on the first postoperative day in group A, while in group B, these values peaked on the second postoperative day and then began to decline. In addition, no significant differences in inflammatory factors during any specific period after treatment were observed between these two groups (*P* > 0.05).

**Table 3:** Changes in the levels of inflammatory markers of patients in the group A and B

item		preOP	POD1	POD2	POD3	POD5	POD7
WBC	A	8.4 ± 1.8	16.7 ± 2.8	15.4 ± 1.9	13.2 ± 2.2	9.7 ± 4.0	8.4 ± 0.9
	B	7.4 ± 2.8	15.9 ± 2.1	16.1 ± 2.6	13.6 ± 2.0	10.8 ± 1.6	8.4 ± 0.9
	*P*	0.08	0.38	0.12	0.41	0.10	0.81
PCT	A	0.36 ± 0.15	9.24 ± 3.34	8.91 ± 1.55	4.84 ± 1.22	0.77 ± 0.18	0.41 ± 0.2
	B	0.32 ± 0.09	8.37 ± 1.08	9.42 ± 0.90	5.57 ± 2.06	0.81 ± 0.10	0.44 ± 0.1
	*P*	0.14	0.1	0.07	0.06	0.23	0.37
CRP	A	0.76 ± 0.7	58.6 ± 28.2	127 ± 27.8	41.9 ± 34.6	17.4 ± 9.3	4.70 ± 2.9
	B	1.2 ± 1.3	68.7 ± 26.7	133 ± 25.5	57.2 ± 43.3	20.3 ± 8.5	5.40 ± 3.8
	*P*	0.11	0.10	0.26	0.08	0.13	0.34
IL-6	A	2.9 ± 1.4	189 ± 25.8	66.8 ± 22.0	27.3 ± 26.6	10.1 ± 8.2	8.3 ± 1.6
	B	3.4 ± 1.1	196 ± 21.3	72.9 ± 18.7	37.3 ± 27.4	15.1 ± 14.7	10.2 ± 6.7
	*P*	0.10	0.17	0.18	0.10	0.07	0.10

A = group A, B = group B

## Discussion

The traditional surgery for VSD is performed with cardiac arrest under CPB [3]. In our cardiac center, a median incision or a right infra-axillary incision is typically chosen. Our surgical teams found no statistically significant differences in the time of CPB, aortic clamping, operative time or prognoses between the median incision and the infra-axillary incision [4]. Many articles describe the advantages and disadvantages of transcatheter device closure of VSD [13, 14]. In addition to these two methods, transthoracic device closure guided by echocardiography is a recently described alternative to surgery. Due to the specific advantages of such a procedure, patients are more likely to accept transthoracic device closure than conventional surgery, especially in China [5–7, 12].

Stimulation of cardiac surgery can induce the synthesis and release of inflammatory cytokines, causing a systemic inflammatory response [15]. The systemic inflammatory response involves the interaction of a large number of cytokines, inflammatory mediators and inflammatory cells. WBC count is one of the classic clinical methods used to diagnose a bacterial infection. Although the medical reference range of the WBC count is relatively wide and clear interindividual differences exist, WBC count was still included in this study since it is the most commonly used parameter. CRP is a typical acute phase protein that is expressed in acute inflammation, but its levels are extremely low in the blood of healthy people. CRP is synthesized and secreted by hepatocytes under stress, which activates complement through the classic pathway, thus participating in systemic inflammation [16]. PCT is the precursor of calcitonin, and it is produced by the expression of the calcitonin I gene on chromosome 11 and inhibited in the absence of infection [17, 18]. Therefore, PCT has unique advantages in the identification of infectious or noninfectious inflammatory responses and has been reported to be more reliable than traditional indicators such as WBC count, CRP and other markers in distinguishing infection and noninfection [19–21]. IL-6 is an important inflammatory mediator and is produced by a variety of cells, including activated T cells and B cells, mononuclear macrophages, and endothelial cells. Studies have shown that IL-6 production is associated with surgery and that patients undergoing cardiac surgery experience a similar increase in IL-6 regardless of whether CPB is performed, similar to the results of this study [22].

A study by Güvener M. et al. showed that the severity of the systemic inflammatory response was significantly related to the time of CPB and aortic clamping [23]. Madhok A.B. et al. also demonstrated changes in postoperative inflammatory markers, confirming that CPB and aortic clamping were major factors in the systemic inflammatory response [24]. Moreover, many scholars have proven that inflammatory factors increase notably after traditional surgery under CPB [8, 25, 26]. In this study, we primarily compared transthoracic device closure of VSD with conventional surgical repair of VSD under CPB with regard to the operative time, mechanical ventilation time, intensive care unit time, drainage, and incision length. As shown in Table 2, we clearly demonstrated that the transthoracic method is superior to traditional surgery, which is the same conclusion that has been reached by other researchers. We hypothesize that such advantages of the transthoracic method may be due to avoiding CPB and cardiac arrest. No studies have reported whether this procedure causes a systemic reaction or whether the inflammatory responses caused by surgery are more severe than those of the transthoracic method.

Based on the results of this study, the transthoracic approach resulted in a clear systemic inflammatory response. Furthermore, no significant difference in the systemic inflammatory response was found between the transthoracic device closure group and the conventional surgery group. Although many studies have shown that transthoracic device closure is less invasive than traditional procedures and has irreplaceable advantages, it is still associated with a significant systemic inflammatory response. We speculated that the underlying reasons may include the following aspects: the pericardium still needs to be opened, and the puncture of the right ventricle and the embedded occluder may directly lead to myocardial injury. In addition, blood flow through the occluder may also cause an inflammatory response. Therefore, we need to remain aware of the possible occurrence of a systemic inflammatory response after such a procedure. However, whether the occurrence of these inflammatory factors can be predicted and the prognosis of the systemic inflammatory response need further study.

Miyamoto K. et al. showed that the increase in inflammatory cytokines was related to the prognosis of the patient [27]. Therefore, knowing the changes in and trends of inflammatory factors after defect closure and being aware of the possible occurrence of a systemic inflammatory response are important, as these practices could help guide clinical treatment. In the device group, the systemic inflammatory marker levels peaked on the first postoperative day (POD1), except for CRP, which peaked on the second postoperative day (POD2). Then, CRP slowly decreased and was basically controlled on the fifth postoperative day (POD5). However, in the surgical repair group, inflammatory markers did not peak until the second postoperative day (POD2), except for IL-6, which peaked on the first postoperative day (POD1). Although the time needed for the inflammatory factors to peak varied, no statistically significant differences were found in the levels of inflammatory factors between the two groups at any period after treatment.

This study has some limitations. First, it was a single-center observational study with a small group of patients. In addition, only a few inflammatory markers were selected, which was the limiting factor of this study. Furthermore, the relationship between the elevation in inflammatory markers and systemic inflammatory response after transthoracic device closure needs further study.

## Conclusion

Although transthoracic device closure of VSD seems to be less traumatic and involves better postoperative recovery than traditional surgery, it still elicits the same systemic inflammatory response as traditional surgery. Therefore, we should still pay attention to the systemic inflammatory response after transthoracic device closure of VSD.

## Abbreviations

CHD: Congenital heart disease
CPB: Cardiopulmonary bypass
CRP: C-reactive protein
IL-6: Interleukin-6
PCT: Procalcitonin
TEE: Transesophageal echocardiography
TTE: Transthoracic echocardiography
VSD: Ventricular septal defect
WBC: White blood cell
